# Comprehensive Cytokine Gene Expression and Multiplex Analysis in Breast Cancer Patients: Insights Into Immune Profiles and Prognostic Factors’ Association

**DOI:** 10.7759/cureus.85288

**Published:** 2025-06-03

**Authors:** Anisha Raju, Karuvaje Thriveni, Shankaranand S Bharatnur

**Affiliations:** 1 Biochemistry, Kidwai Memorial Institute of Oncology, Bangalore, IND; 2 Pathology, Kidwai Memorial Institute of Oncology, Bangalore, IND

**Keywords:** b-cell lymphoma-6, breast cancer, cytokines, immune profiling, interleukin-6, multiplex assay, myeloid cell leukemia-1, tumor microenvironment

## Abstract

Background: Cytokines are small, soluble proteins secreted by various cells. They are the key mediators of inflammation and immune modulation and play crucial roles in tumor progression, immune evasion, and therapeutic resistance. Cytokines play a pivotal role in breast cancer, influencing tumor initiation, progression, and metastasis. This study aimed to perform an integrative multiplex cytokine profiling and subsequent tissue cytokine expression (gene and protein) analysis to identify immune signatures and potential biomarkers associated with breast cancer progression and clinical parameters.

Methods: A prospective case-control study involving 90 breast cancer patients and 60 age-matched healthy controls was conducted. Peripheral blood was collected for a Luminex-based multiplex assay to analyze 17 cytokines (pro-inflammatory and anti-inflammatory cytokines). Tumor tissue samples were used to assess the gene expression of IL-6 and IL-23 by quantitative real-time polymerase chain reaction (qRT-PCR). Tissue expression of downstream signaling targets such as B-cell lymphoma-6 (BCL-6) and myeloid cell leukemia 1 (MCL-1) was also assessed. Expression levels of pro-inflammatory and immunosuppressive cytokines were evaluated and correlated with clinicopathological features, including tumor stage, grade, hormone receptor status, and menopausal state.

Results: Breast cancer patients demonstrated significantly elevated plasma levels of IL-1β, IL-6, IL-8, IL-12 (p40), granulocyte colony-stimulating factor (G-CSF), tumor necrosis factor-alpha (TNF-α), and monocyte chemoattractant protein 1 (MCP-1) compared to controls. Gene expression of IL-6 and IL-23 was markedly upregulated in tumor tissues and significantly associated with advanced stage (P < 0.001), grade 3 histology (P < 0.001), and hormone receptor-positive status (P = 0.023). Downstream targets BCL-6 and MCL-1 showed increased expression, correlating with disease aggressiveness (advanced stage and high tumor grade, P < 0.001). MCL-1 protein expression, evaluated by immunohistochemistry, was significantly associated with advanced pathological stage (P = 0.005), post-menopausal status (P < 0.001), and lymph node involvement (P = 0.003). Cytokine intercorrelation analysis suggested a complex immune network, indicating that cytokine expression may influence or reflect broader immune dynamics within the tumor microenvironment.

Conclusion: This study highlights distinct cytokine expression profiles in breast cancer, with IL-6, MCP-1, and MCL-1 emerging as potential biomarkers for disease progression and molecular subtype differentiation. These cytokines hold potential as both prognostic biomarkers and therapeutic targets. Future studies incorporating spatial technologies and functional assays will be crucial in translating these findings into clinically actionable strategies for precision oncology.

## Introduction

Breast cancer remains a major global health concern, with an incidence of 2,310,051 new cases globally, at a mortality rate of 17.1 per 100,000 cases [1,2]. Despite considerable progress in early detection, molecular characterization, and therapeutic strategies, the clinical management of breast cancer is complicated by its heterogeneous nature and the complex interactions between tumor cells and the surrounding microenvironment. Increasing evidence suggests that the progression of breast cancer is not merely governed by intrinsic genetic alterations but is profoundly shaped by extrinsic factors such as the tumor microenvironment (TME) [3]. The TME, composed of immune cells, fibroblasts, and extracellular matrix, interacts with cancer cells to influence progression. Among these, cytokines, small, secreted proteins that modulate immune responses, inflammation, and cellular communication, play a pivotal role in driving oncogenic processes, including angiogenesis, epithelial-mesenchymal transition (EMT), and metastasis. Dysregulated cytokine networks are often associated with immune evasion, resistance to treatment, and unfavorable clinical outcomes, thus highlighting their potential utility as biomarkers and therapeutic targets in breast cancer [4].

The TME is a dynamic milieu composed of immune cells, stromal elements, extracellular matrix components, and signaling molecules that collectively influence tumor biology. In breast cancer, cytokines are key orchestrators of immune modulation and tumor progression. Pro-inflammatory cytokines such as interleukin (IL)-6, IL-1β, IL-8, and tumor necrosis factor-alpha (TNF-α) are frequently upregulated and contribute to tumor cell proliferation, migration, and invasion [5]. Conversely, immunosuppressive cytokines like IL-10 and transforming growth factor-beta (TGF-β) inhibit cytotoxic immune responses and facilitate immune escape mechanisms, thereby promoting tumor growth and metastasis. The intricate balance between pro- and anti-inflammatory cytokines is crucial in determining the immunological tone of the TME and, consequently, the behavior of the tumor. Several studies have demonstrated that specific cytokine profiles are associated with distinct molecular subtypes of breast cancer and correlate with prognosis, suggesting their relevance in stratifying patients and tailoring treatment strategies [5,6].

Traditional approaches to cytokine profiling, including enzyme-linked immunosorbent assay (ELISA) and quantitative reverse transcription polymerase chain reaction (qRT-PCR), often rely on bulk tissue analysis, thereby overlooking the complex cytokine milieu in TMEs. To address the need for high-throughput and multiplexed cytokine analysis, we employed the Multiplex Luminex Assay (Merck Millipore, Burlington, MA), which enables simultaneous quantification of multiple cytokines in a single sample. This bead-based immunoassay integrates the sensitivity of ELISA with the efficiency of flow cytometry, offering a robust platform for comprehensive cytokine profiling. Although it does not provide spatial resolution, the Luminex assay is highly valuable for detecting subtle changes in cytokine expression patterns across different experimental conditions. Its ability to process multiple analytes concurrently with minimal sample volume makes it an ideal tool for evaluating the systemic immune response, tumor-associated cytokine profiles, and potential biomarkers for disease progression and therapeutic response [7].

This study aims to comprehensively characterize the cytokine landscape in breast cancer by integrating transcriptomic (qRT-PCR) and proteomic (Luminex bead-based multiplex assay) data. This dual-platform approach facilitates an in-depth assessment of cytokine expression at both the gene and protein levels, capturing the complexity of immune signaling in the TME. The objectives of the study are to identify cytokine signatures that correlate with clinical parameters such as tumor grade, molecular subtype, and response to therapy; to differentiate cytokine expression patterns across various subtypes of breast cancer; and to explore potential cytokine-based biomarkers that may enhance diagnostic and prognostic accuracy.

## Materials and methods

Study design

This study was designed as a prospective case-control investigation to evaluate cytokine gene expression and multiplex cytokine profiling in breast cancer patients compared to healthy controls. Institutional ethical approval (Kidwai Memorial Institute of Oncology; dated: 1-3-2010) was obtained before study initiation, and written informed consent was obtained from all participants. The sample collection was conducted from January 1, 2014, to December 31, 2016. All study procedures followed the ethical principles of the Declaration of Helsinki. Participant data confidentiality was rigorously maintained, and all data collection and sample handling procedures followed strict ethical and biosafety standards.

Study population

Using Cochran’s formula with a 5% significance level and 1% margin of error, the minimum estimated sample size was 96 breast cancer patients. A total of 90 female patients with histologically confirmed breast cancer and 60 age-matched healthy female controls were enrolled from the oncology department of a regional cancer institute in India. The control group consisted of individuals undergoing routine health evaluations with no history of malignancy, autoimmune conditions, or chronic inflammatory diseases. Eligible patients were between the ages of 30 and 70 years and had not received chemotherapy, radiotherapy, or immunotherapy before sample collection. Exclusion criteria included concurrent malignancies, chronic infections, autoimmune diseases, those undergoing immunosuppressive treatment, and pregnancy or lactation.

Clinical features

Breast cancer patients were categorized according to clinical staging based on the American Joint Committee on Cancer (AJCC) TNM (tumor, node, and metastasis) classification system (stages I-IV). Further molecular classification was performed using immunohistochemical (IHC) profiling for estrogen receptor (ER), progesterone receptor (PR), and fluorescence in situ hybridization (FISH) for HER2/neu status. Patients were accordingly grouped into molecular subtypes: luminal A (ER+, PR+, HER2−, low Ki67), luminal B (ER+, PR+, HER2+ and high Ki-67), HER2-enriched (ER−, PR−, HER2+), and triple-negative breast cancer (TNBC) (ER−, PR−, HER2−).

Sample collection

Peripheral venous blood samples (5 mL) were collected in ethylenediaminetetraacetic acid (EDTA)-coated tubes from all participants and processed within 30 minutes. Blood samples from cancer patients were collected one day prior to the surgery, while those from controls were collected in the morning. Plasma was separated by centrifugation at 3,000 rpm for 10 minutes at 4°C and aliquoted for storage at -80°C until cytokine quantification. Tumor tissue samples (~30 mg) were obtained from surgical specimens and stored in RNA preservation solution (RNAlater) at −80°C immediately. Adjacent grey-white non-tumorous breast tissue was collected for comparative analysis. The DNA extraction and genotyping were conducted according to a previous publication by Thriveni et al. [8].

Multiplex assay

The cytokine multiplex assay (Merck Millipore, Burlington, MA) was a screening test conducted to detect 17 cytokines in the plasma of breast cancer patients and healthy female controls. The assay contained dyed beads conjugated with monoclonal antibodies specific for each cytokine. The antibody-conjugated beads are allowed to react with a sample and a secondary or detection antibody in a microplate well to form a capture-type “sandwich” immunoassay. Multiplex assays can be created by mixing bead sets with different conjugated antibodies to test simultaneously for many analytes in one sample. The following cytokines were analyzed: interferon-gamma (IFN-γ), IL-1β, IL-2, IL-4, IL-5, IL-6, IL-7, IL-8, IL-10, IL-12 (p40), IL-13, IL-17, monocyte chemoattractant protein 1 (MCP-1), granulocyte colony-stimulating factor (G-CSF), granulocyte-macrophage colony-stimulating factor (GM-CSF), and tumor necrosis factor (TNF-α and TNF-β) in a single microplate well.

Gene expression analysis

Following a previously published protocol [8], total RNA was isolated from tumor tissue using the TRIzol reagent (Invitrogen, Waltham, MA). RNA integrity and purity were assessed using a NanoDrop spectrophotometer, ensuring an A260/A280 ratio of ≥1.8 for downstream applications. Complementary DNA (cDNA) synthesis was performed from 1 μg of RNA using the High-Capacity cDNA Reverse Transcription Kit (Applied Biosystems, Waltham, MA). qRT-PCR was conducted using the Eppendorf Mastercycler® ep realplex (2S; Eppendorf, Hamburg, Germany) and SYBR Green Master Mix (Qiagen, Hilden, Germany). Primer sequences for cytokine genes were designed via the National Center for Biotechnology Information (NCBI) Primer-BLAST and optimized for amplification efficiency. Target genes included IL-6, IL-23, B-cell lymphoma-6 (BCL-6), and myeloid cell leukemia-1 (MCL-1), with hypoxanthine-guanine phosphoribosyltransferase (HPRT) and beta-2-microglobulin (β2M) serving as housekeeping controls (Supplementary Table A1). Relative gene expression was calculated using the 2^−ΔΔCt^ method.

Cytokine immunohistochemistry analysis

The MCL-1 protein expression analysis was performed on formalin-fixed paraffin-embedded invasive ductal carcinoma breast tissue blocks arranged on six tissue microarray slides. Cores of 0.6 mm diameter represented each case, spaced 0.8 mm apart. The paraffin-embedded tissue sections (3-4 μm) were mounted on silane-coated slides, deparaffinized in xylene, rehydrated through graded ethanol, and subjected to antigen retrieval using Tris-EDTA buffer (pH 9.0) in a pressure cooker. Endogenous peroxidase activity was blocked with 3% H₂O₂, followed by incubation with MCL-1 primary antibodies, and detection using a Biogenix (Lucknow, India) secondary antibody kit with DAB (3,3'-diaminobenzidine) as chromogen. Sections were counterstained with hematoxylin and mounted in DPX (dibutylphthalate polystyrene xylene). Negative controls were processed identically, excluding the primary antibody. The percentage of stained tumor cells was scored with precision as 0 (negative), 1 (<10%), 2 (10%-50%), and 3 (>50%). Staining intensity was scored with accuracy as 0 (negative), 1 (mild), 2 (moderate), and 3 (strong). A normal lung tissue section stained with MCL-1 served as a positive control (as suggested by the manufacturer). The section with no primary antibody served as the negative control.

Statistical method

To ensure analytical rigor, cytokine data were log-transformed where necessary to normalize distributions. There was no data imputation, and out layers were removed from the analysis. Statistical analyses were conducted using IBM SPSS software (version 27.0, IBM Corp., Armonk, NY). Descriptive statistics were calculated. Since the cytokine levels did not follow normal distribution, a non-parametric test (Mann-Whitney U test) was used to associate the cytokine levels between the patient and control groups. Correlation between the variables (cytokines) was evaluated using the Spearman correlation coefficient after log transformation. Spearman’s rank correlation coefficients were computed to explore associations between cytokine expression and clinical variables, including tumor size and lymph node status. Multivariate regression models were employed to identify independent predictors of altered cytokine expression.

The statistical analysis between the level of gene expression of cytokines IL-6, IL-23, and downstream targets MCL-1 and BCL-6 with the clinicopathological parameters was done by Pearson’s chi-square and Fisher's exact test by using Graphpad Prism version 5.00 (GraphPad Software, San Diego, CA). A P-value of less than 0.05 was considered statistically significant.

## Results

Demographic and clinicopathological characteristics of the study population

The median age of breast cancer patients was 46 years (range: 39-56), while the control group had a median age of 49.5 years (range: 35-62), with no statistically significant difference observed (P > 0.05). The majority of patients (69%) were above the age of 40 at diagnosis, and 55% were post-menopausal. Histopathological analysis revealed that 80% of the tumors were of grade 3, and 71% of the cases exhibited lymph node metastasis. Immunohistochemical characterization showed that 52% of tumors were estrogen receptor (ER) positive, 45% were progesterone receptor (PR) positive, and 34% showed HER2/neu overexpression.

Plasma cytokine profiling and differential expression

Multiplex bead-based analysis of plasma samples revealed a distinctive cytokine profile between breast cancer patients and healthy controls. While the control group generally exhibited low baseline levels of circulating cytokines, breast cancer patients demonstrated significantly elevated concentrations of multiple cytokines, including IL-1β, IL-6, IL-8, IL-12 (p40), IL-13, G-CSF, TNF-α, and MCP-1. Notably, IL-6 and G-CSF levels were profoundly elevated in the patient group (P = 0.003 and P = 0.005, respectively). These findings point toward a pro-inflammatory milieu associated with breast cancer (Table 1).

**Table 1:** Association of plasma cytokine levels (pg/mL) with control and patient groups. * Significant P < 0.05. ^ A t-test was performed with a t-value of 4.394. The Mann-Whitney U test measured the association between cytokine levels. IFN-γ: interferon-gamma; IL: interleukin; G-CSF: granulocyte colony-stimulating factor; GM-CSF: granulocyte-macrophage colony-stimulating factor; TNF-α: tumor necrosis factor-alpha; tumor necrosis factor-beta; MCP-1: monocyte chemoattractant protein 1; AST: aspartate transaminase; ALT: alanine aminotransferase.

Variables	Controls (pg/mL)			Cases (pg/mL)			P-value
	Min	Max	Median	Min	Max	Median	
Age	24	76	44	25	78	47	0.001*^
IFN-γ	1	13	4	0	31	3	0.016*
IL-1β	0	3	3	0	182	3	0.000*
IL-2	0	5	3	0	19	3	0.003*
IL-4	0	16	3	3	88	3	0.389
IL-5	0	6	1	0	24	2	0.225
IL-6	0	13	3	0	1391	3	0.003*
IL-7	0	28	4	0	54	3	0.058
IL-8	2	1165	13	1	2169	8	0.194
IL-10	0	13	3	0	66	3	0.940
IL-12 (p40)	0	30	5	0	102	3	0.045*
IL-13	1	9	3	2	68	3	0.028*
IL-17	0	6	3	0	88	3	0.054
G-CSF	9	193	28	3	313	60	0.005*
GM-CSF	2	38	8	0	89	7	0.387
TNF-α	0	78	7	0	195	3	0.092
TNF-β	0	6	3	0	19	3	0.215
MCP-1	79	865	324	3	1422	288	0.445
AST/ALT	0	3	1	0	6	1	0.214

Among the pro-inflammatory cytokines, IL-1β, IL-6, and IL-12 showed the most robust differences, with IL-1β showing a maximum plasma concentration of 182 pg/mL in cancer patients, compared to 3 pg/mL in controls. Similarly, G-CSF levels reached up to 313 pg/mL in cases versus 193 pg/mL in controls. Interestingly, IL-8, which was elevated in both the control (1165 pg/mL) group and patient samples (2169 pg/mL), exhibited a nearly 2-fold increase in cancer patients. The elevation in the cancer patients suggests a systemic activation of innate immune pathways associated with tumor-driven inflammation.

Association between cytokine levels and clinicopathological parameters

Correlation analysis between plasma cytokine levels and clinicopathological features revealed that elevated levels of IFN-γ, IL-1β, IL-2, IL-6, and IL-12 (p40) were significantly associated with advanced clinical stages of breast cancer (P < 0.05). IL-5 and TNF-β were also significantly associated with higher histological grades (P = 0.04 and P = 0.045, respectively). However, none of the plasma cytokines significantly correlated with ER, PR, or HER2/neu status. Similarly, no significant association was found between patient age and aspartate transaminase (AST)/alanine aminotransferase (ALT) liver enzyme levels (Table 2).

**Table 2:** Cytokine correlation with clinicopathological parameters (Spearman Rho correlation coefficient). * Significant P < 0.05. ** Significant P < 0.01. The correlation between cytokines and clinicopathological parameters was established by the Spearman rank correlation coefficient analysis (ρ). IFN-γ: interferon-gamma; IL: interleukin; G-CSF: granulocyte colony-stimulating factor; GM-CSF: granulocyte-macrophage colony-stimulating factor; TNF-α: tumor necrosis factor-alpha; tumor necrosis factor-beta; MCP-1: monocyte chemoattractant protein 1; ER: estrogen receptor; PR: progesterone receptor.

		IFN-γ	IL1-β	IL-2	IL-4	IL-5	IL-6	IL-7	IL-8	IL-10	IL-12	IL-13	IL-17	G-CSF	GM-CSF	TNF-α	TNF-β	MCP-1
Age	Correlation coefficient (ρ)	.058	.085	.016	.016	.041	.074	.015	.202^*^	.195	.190	.135	.187	-.156	.053	.213^*^	-.031	.057
	Sig. (2-tailed)	.576	.412	.881	.879	.695	.479	.883	.049	.058	.065	.193	.070	.132	.609	.038	.769	.584
Grade	Correlation coefficient (ρ)	-.056	.074	-.029	-.092	.210^*^	.113	.056	.068	.185	-.066	-.044	-.070	.109	.115	.119	-.201^*^	.114
	Sig. (2-tailed)	.586	.473	.780	.374	.040	.273	.591	.512	.071	.521	.669	.498	.290	.266	.250	.049	.268
ER	Correlation coefficient (ρ)	-.148	-.203	-.149	-.036	-.089	-.233	-.104	-.097	-.190	-.236	-.210	-.030	.020	-.114	-.089	-.120	-.199
	Sig. (2-tailed)	.271	.129	.268	.790	.510	.081	.443	.471	.156	.078	.117	.823	.881	.396	.509	.375	.138
PR	Correlation coefficient (ρ)	.014	-.232	-.036	-.048	-.120	-.215	-.009	-.019	-.059	-.100	-.126	.051	.068	-.110	-.059	-.109	-.199
	Sig. (2-tailed)	.918	.082	.791	.721	.373	.108	.945	.889	.664	.460	.351	.707	.618	.417	.662	.420	.137
HER2	Correlation coefficient (ρ)	.043	-.026	-.017	-.121	-.192	.094	.011	-.219	.069	-.033	.207	.122	-.066	-.178	-.037	.180	-.195
	Sig. (2-tailed)	.748	.847	.897	.371	.153	.485	.934	.102	.611	.808	.123	.367	.625	.185	.784	.179	.146
Tumor stage	Correlation coefficient (ρ)	.279^**^	.264^**^	.299^**^	.178	.122	.301^**^	.057	-.043	-.038	.227^*^	.129	.054	-.159	-.158	-.006	-.117	-.044
	Sig. (2-tailed)	.006	.009	.003	.083	.235	.003	.579	.680	.715	.026	.253	.636	.121	.124	.953	.258	.667

Cytokine intercorrelation analysis

Spearman correlation analysis among cytokines revealed strong interrelationships between several pro-inflammatory and regulatory cytokines. Notable positive correlations were observed between IFN-γ, IL-1β, IL-4, IL-5, IL-6, IL-8, IL-10, TNF-α, IL-12 (p40), G-CSF, and GM-CSF (Supplementary Table A2). These findings indicate a possible cascade-like cytokine activation in the tumor microenvironment, wherein the induction of one cytokine potentially triggers the upregulation or suppression of others. Such a networked cytokine landscape underscores the complexity of immune modulation in breast cancer.

Tissue cytokine gene expression and downstream pathway activation

To validate plasma cytokine findings at the tissue level, qRT-PCR was performed on tumor tissue specimens for IL-6 and IL-23 gene expression. Results demonstrated a 4.6-fold increase in IL-6 and a 7.4-fold increase in IL-23 mRNA expression in tumor tissues compared to adjacent non-tumorous tissues (P < 0.001 and P < 0.0001, respectively). This supports the systemic and local pro-inflammatory activation in breast cancer.

**Figure 1:**
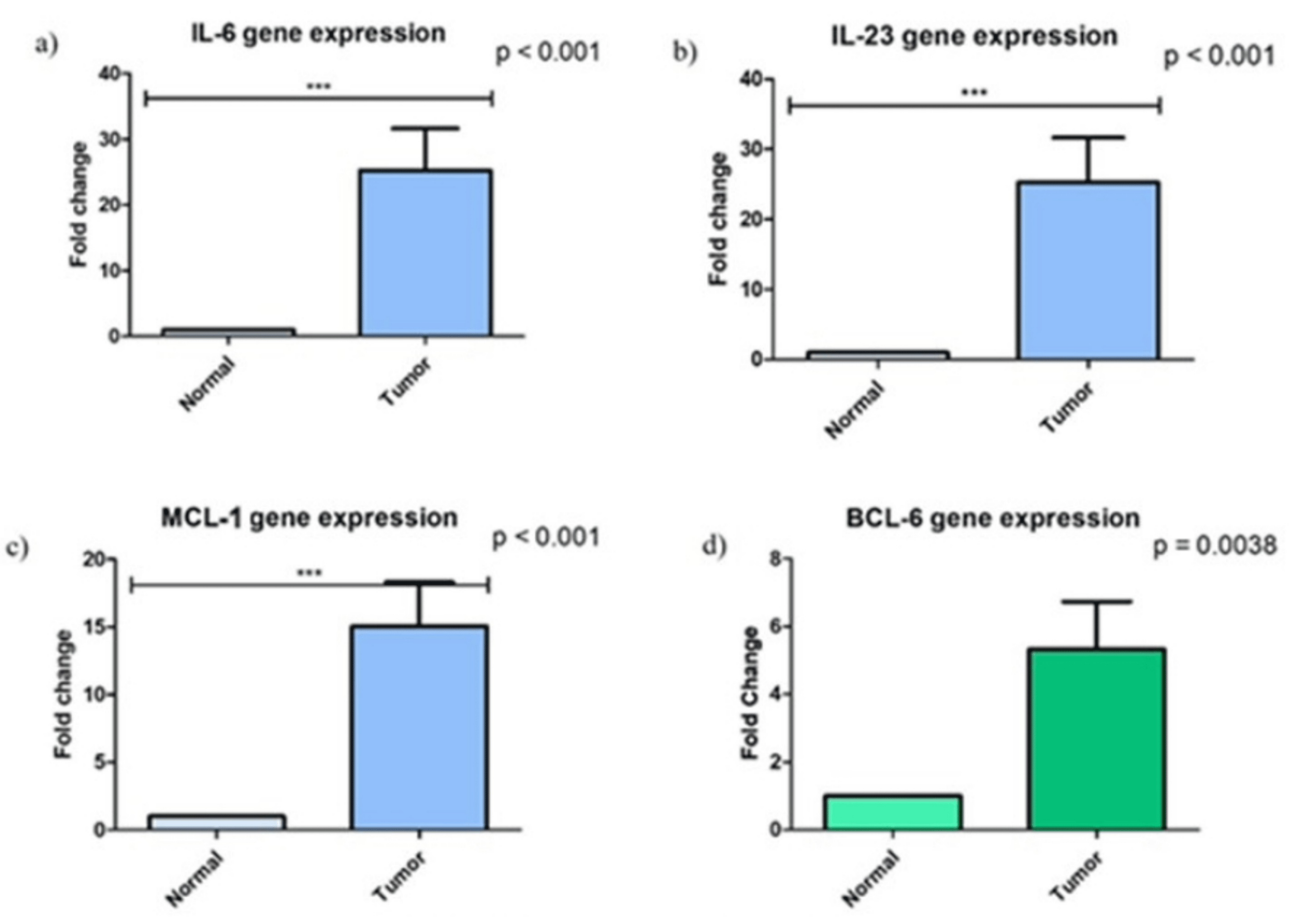
Gene expression analysis of (a) IL-6, (b) IL-23, (c) MCL-1, and (d) BCL-6 by RT-PCR in breast cancer tissues and the fold change in comparison to normal tissue. IL: interleukin; MCL-1: myeloid cell leukemia 1; BCL-6: B-cell lymphoma-6; RT-PCR: reverse transcription polymerase chain reaction.

Further downstream analysis revealed that the pSTAT3 signaling pathway was activated in breast cancer tissues. Expression of anti-apoptotic gene MCL-1 and differentiation marker BCL-6, downstream targets of the IL-6/IL-23/pSTAT3 axis, was significantly higher in tumor samples, with 1.5-fold and 3.5-fold increase in tumor tissues compared to the adjacent non-tumorous tissue (P = 0.0038 and P < 0.001, respectively). These markers suggest enhanced cell survival and alter immune differentiation within the tumor microenvironment (Figure 1).

Correlation of gene expression with clinicopathological parameters

**Figure 2:**
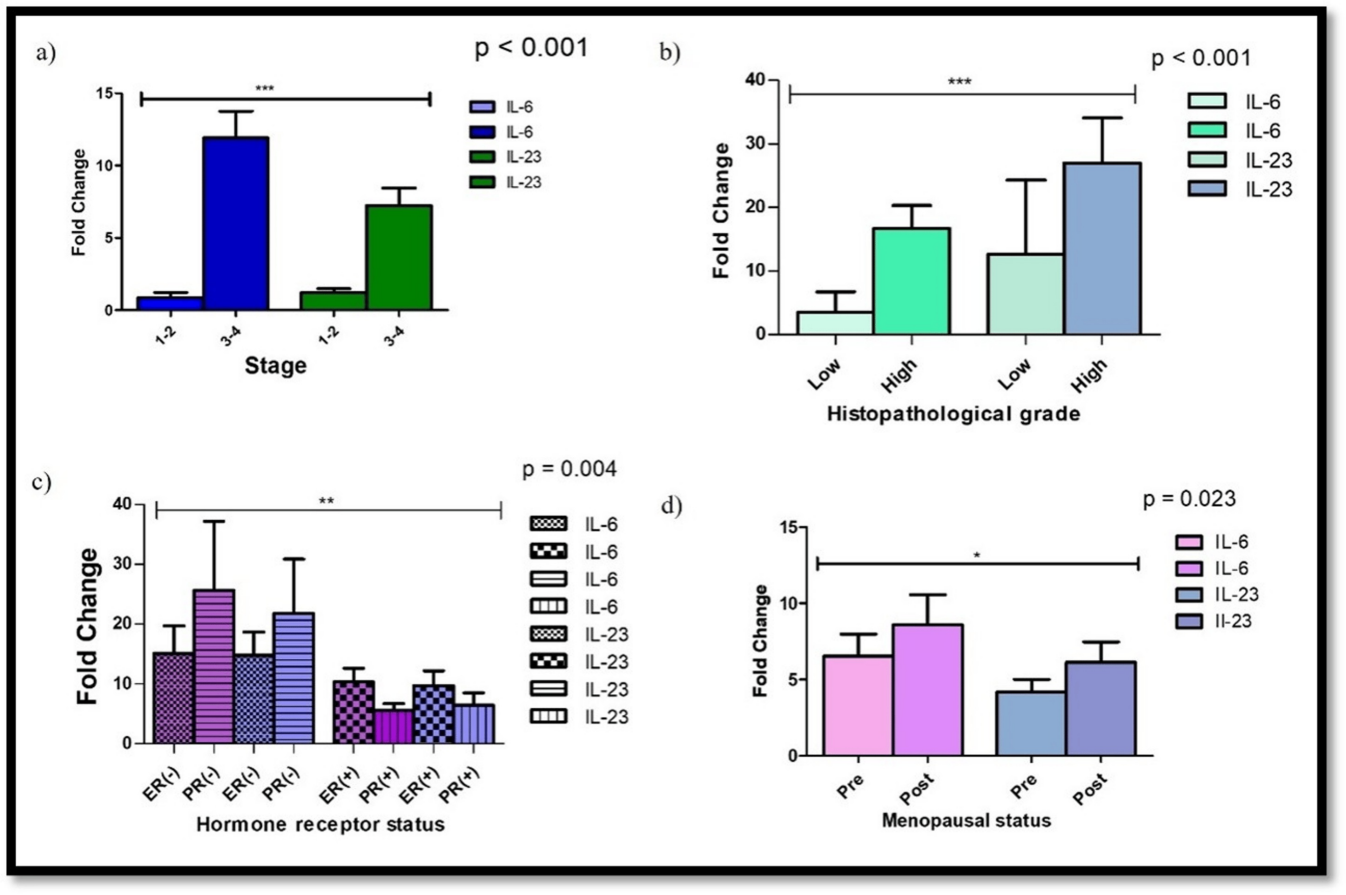
Correlation between the gene expression of IL-6 and IL-23 and clinicopathological parameters: (a) stage of disease, (b) histopathological grade, (c) hormone receptor status, and (d) menopausal status. ER: estrogen receptor; PR: progesterone receptor; IL: interleukin.

Elevated gene expression of IL-6 and IL-23 was significantly associated with advanced tumor stage (P < 0.001), histological grade 3 (P < 0.001), and post-menopausal status (P = 0.023). Interestingly, a positive correlation was also found with hormone receptor status (ER and PR positive) (P = 0.004), although no significant correlation was seen with HER2/neu status or lymph node involvement (Figure 2).

**Figure 3:**
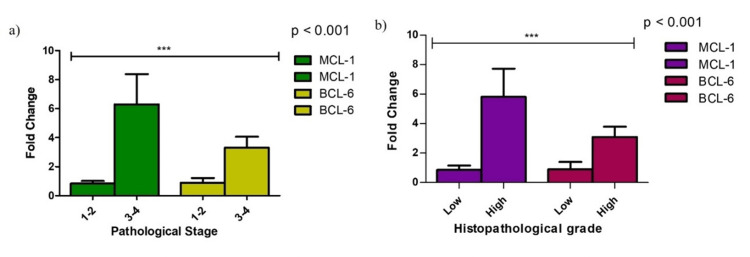
Correlation between the gene expression of MCL-1 and BCL-6 and clinicopathological parameters: (a) stage of disease and (b) histopathological grade. * Significant P < 0.05. MCL-1: myeloid cell leukemia 1; BCL-6: B-cell lymphoma-6.

MCL-1 and BCL-6 gene expression showed significant associations with advanced stage and high tumor grade (P < 0.001), but not with menopausal status or hormone receptor status (P > 0.05) (Figure 3). These data highlight the role of the IL-6/IL-23 axis in promoting aggressive tumor behavior and resistance to apoptosis, particularly in advanced stages.

MCL-1 protein expression by immunohistochemistry

**Figure 4:**
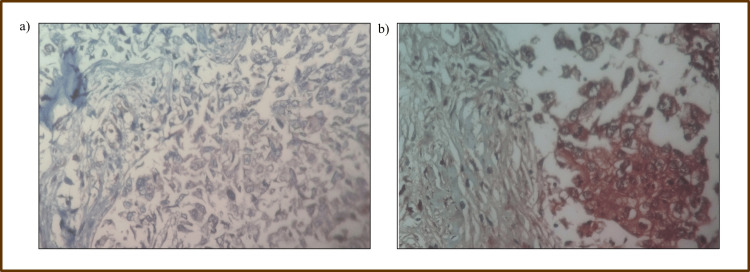
Immunohistochemistry staining for anti-apoptotic marker MCL-1 on invasive ductal carcinoma breast tissue displaying cytoplasmic and membranous staining patterns: (a) negatively stained cells and (b) positively stained cells. MCL-1: myeloid cell leukemia 1.

Immunohistochemical staining of MCL-1 protein was performed using tissue microarrays derived from formalin-fixed, paraffin-embedded breast cancer tissues. MCL-1 expression was categorized as negative, low, intermediate, or high. High MCL-1 expression was observed in 57% of tumor samples, with intermediate expression in 28% (Figure 4).

Statistical analysis revealed a significant association between high MCL-1 expression and advanced pathological stage (P = 0.005), post-menopausal status (P < 0.001), and lymph node involvement (P = 0.003). However, MCL-1 protein levels did not significantly correlate with histological grade or hormone receptor status (Table 3).

**Table 3:** Association of MCL-1 protein expression with clinicopathological parameters. * Significant P < 0.05. Pearson’s chi-square (χ2) test evaluated the association between protein expression of MCL1 and clinicopathological parameters. MCL-1: myeloid cell leukemia 1.

Markers	Menopausal status	Pathological stage	Lymph node status	Hormone receptor status	Histopathological grade
MCL-1	<0.001	0.005	0.003	0.949	0.516

## Discussion

Breast cancer remains the most frequently diagnosed cancer among women globally, contributing significantly to cancer-related morbidity and mortality [2,9]. Despite growing awareness and advancements in detection and therapeutic strategies, the clinical course of breast cancer remains unpredictable due to its molecular and immunological heterogeneity [1]. Our study aimed to explore this heterogeneity by comprehensively evaluating cytokine gene expression and multiplex cytokine profiling in breast cancer patients to identify potential immune biomarkers associated with disease progression and subtype-specific immune modulation.

Our findings demonstrate a significant upregulation of key pro-inflammatory cytokines such as IL-6, IL-1β, TNF-α, and IL-12 (p40) in the plasma of breast cancer patients, with expression correlating positively with tumor stage and grade.

IL-6 emerged as a particularly important cytokine, significantly overexpressed in both the plasma and tumor tissue of patients, especially those with advanced-stage or TNBC. This aligns with recent literature emphasizing the role of IL-6 in activating STAT3 signaling, which in turn drives oncogenic pathways, including proliferation, survival, and stemness of cancer cells [10]. Our study also found elevated expression of pSTAT3 downstream targets such as BCL-6 and MCL-1, suggesting a functionally active IL-6/STAT3 signaling axis in high-grade tumors. Notably, MCL-1 protein overexpression was confirmed via immunohistochemistry and was significantly associated with advanced pathological stage and lymph node involvement, further supporting its role in cancer cell survival and resistance to apoptosis.

The observed cytokine intercorrelation patterns indicate the existence of complex regulatory feedback loops within the TME. The synchronous elevation of multiple pro-inflammatory and immunosuppressive cytokines, including IL-10 and TGF-β, suggests that breast tumors may establish a paradoxical immune contexture, simultaneously promoting inflammation to support tumor growth and suppressing effective anti-tumor immunity. This dualistic immune profile is consistent with prior spatial and multiplex analyses of breast and melanoma tumors, demonstrating the co-localization of immunostimulatory and inhibitory molecules within distinct tumor niches [11,12].

Importantly, our results demonstrate that cytokine profiles differ across molecular subtypes. For instance, IL-10 and TGF-β were more commonly elevated in luminal subtypes, aligning with their known roles in regulatory T cell (Treg) recruitment and immune suppression [13]. In contrast, the TNBC subgroup showed a markedly heightened expression of IL-6 and MCP-1, both at the gene and protein levels, reinforcing their potential as biomarkers for aggressive breast cancer phenotypes. These findings not only corroborate existing work on immune subtype characterization [14,15] but also propose specific cytokines as candidates for risk stratification and targeted therapy.

The spatial context of cytokine signaling in breast cancer is increasingly recognized as a determinant of immunotherapy response. Although this study utilized bulk qRT-PCR and plasma multiplex assays, our results complement emerging spatial technologies like imaging mass cytometry (IMC), which have revealed that cytokines and their downstream effectors often exhibit spatial compartmentalization that dictates immune infiltration and therapeutic response [16,17]. For example, MCL-1 expression localized to stromal regions has been associated with poor outcomes, echoing our findings of its correlation with advanced disease stage.

These insights underscore the importance of multidimensional immune profiling in breast cancer. While traditional assays provide valuable quantitative data, integrating them with complementary high-throughput platforms could enhance biomarker discovery and inform patient-specific therapeutic strategies [18,19]. Although spatially resolved platforms like IMC have shown promise in identifying immunotherapy responders across cancers, including melanoma and breast cancer [20,21], our study employed the Multiplex Luminex Assay to quantify key cytokines, such as IL-6 and IL-23, which may represent critical nodes in immune regulation within the tumor microenvironment.

The Multiplex Luminex Assay represents a significant advancement over traditional single-plex assays, enabling simultaneous quantification of multiple cytokines from limited sample volumes with high sensitivity and reproducibility [7]. While it does not provide spatial context, this platform offers robust insights into the immune milieu and cytokine-driven signaling pathways in tumors. In our study, the combined use of qRT-PCR and multiplex bead-based assays provided a comprehensive view of cytokine expression patterns across samples. These findings contribute to understanding immune modulation in breast cancer and lay the groundwork for future investigations that may incorporate spatial profiling to map these cytokines to specific tumor and stromal compartments. Ultimately, this integrative approach can support more informed biomarker discovery and tailored therapeutic strategies, particularly in immune-evasive cancers like triple-negative breast cancer.

Our study contributes to the ongoing effort to decipher the immunobiological underpinnings of breast cancer. The strong association between cytokine expression and clinicopathological variables such as tumor grade, lymph node involvement, and hormone receptor status highlights the potential of cytokine profiling in improving prognostication. Furthermore, identifying cytokine-specific expression patterns across molecular subtypes opens avenues for personalized medicine, where targeted modulation of the immune microenvironment could complement conventional treatments [22,23].

However, some limitations of this study should be acknowledged. While the multiplex assay offers significant advantages in terms of sensitivity, efficiency, and simultaneous detection of multiple cytokines, it is not without limitations. Some key limitations include inter-assay variability, potential cross-reactivity, and differences in sensitivity across targets. These factors may affect data accuracy and require careful standardization.

The study's cross-sectional design precludes longitudinal assessment of cytokine dynamics over the course of disease progression or treatment. Although we provide molecular and immunohistochemical evidence, the functional impact of cytokine modulation remains to be elucidated through mechanistic studies and clinical trials. Additionally, financial constraints restricted the functional validation of cytokines within the tumor microenvironment.

## Conclusions

The present study was unique as the first to explore 17 cytokines (both pro- and anti-inflammatory) in the peripheral blood samples of breast cancer patients, thereby allowing the analysis of multiple cytokines at a single time point. This can serve as a critical test in the preliminary diagnosis of breast cancer and may assist in treatment planning.

Our integrative analysis combining cytokine gene expression and plasma profiling elucidates a complex yet informative immune landscape in breast cancer. IL-6 and MCL-1 emerged as key immunological markers associated with disease progression and subtype differentiation. These cytokines hold potential as both prognostic biomarkers and therapeutic targets. Although not utilized in the present study, spatial transcriptomics represents a promising future approach to complement multiplex cytokine profiling by adding spatial information about cytokine expression in the tumor microenvironment.

## Appendices

Supplementary Table A1

**Table 4:** Primer sequence for cytokine gene expression analysis. FW: forward primer; RV: reverse primer; bp: base pair.

Gene	Primer sequence	Size
Interleukin-6 (IL-6)	FW: 5'-ATGCAATAACCACCCCTGAC-3'	20 bp
	RV: 5'-GAGGTGCCCATGCTACATTT-3'	20 bp
Interleukin-23 (IL-23)	FW: 5'-GCAGATTCCAAGCCTCAGTC-3'	20 bp
	RV: 5'-CCTTGAGCTGCTGCCTTTAG-3'	20 bp
Myeloid cell leukemia- 1 (MCL-1)	FW: 5'-TGCTGGAGTAGGAGCTGGTT-3'	20 bp
	RV: 5'-TCCTCTTGCCACTTGCTTTT-3'	20 bp
B-cell lymphoma-6 (BCL-6)	FW: 5'-CCAAGGAAACAATCCCAGAA-3'	20 bp
	RV: 5'-AAATGCAGGGCAATCTCATC-3'	20 bp
Beta-2-microglobulin (β2M)	FW 5’-GAGTATGCCTGCCGTGTG-3’	18 bp
	RV 5’-AATCCAAATGCGGCATCT-3’	18 bp
Hypoxanthine-guanine phosphoribosyltransferase (HPRT)	FW 5’-TGACACTGGCAAAACAATGCA-3’	21 bp
	RV 5’-GGTCCTTTTCACCAGCAAGCT-3’	21 bp

Supplementary Table A2

**Table 5:** Intercorrelation between the cytokines (Spearman Rho correlation coefficient). * Significant P < 0.05. ** Significant P < 0.01. The intercorrelation between cytokines was established by the Spearman rank correlation coefficient analysis (ρ). IFN-γ: interferon-gamma; IL: interleukin; G-CSF: granulocyte colony-stimulating factor; GM-CSF: granulocyte-macrophage colony-stimulating factor; TNF-α: tumor necrosis factor-alpha; tumor necrosis factor-beta; MCP-1: monocyte chemoattractant protein 1.

		IFN-γ	IL1-β	IL-2	IL-4	IL-5	IL-6	IL-7	IL-8	IL-10	IL-12	IL-13	IL-17	G-CSF	GM-CSF	TNF-α	TNF-β	MCP-1
IFN-γ	Correlation coefficient (ρ)	1.000	.043	.141	.228^*^	.212^*^	.275^**^	.196	.100	.279^**^	.068	.136	.061	.030	.190	.247^*^	.239^*^	-.004
	Sig. (2-tailed)	.	.678	.172	.025	.038	.007	.056	.332	.006	.507	.186	.557	.775	.063	.015	.019	.971
IL1-β	Correlation coefficient (ρ)	.043	1.000	.343^**^	.156	.178	.122	-.078	.066	.264^**^	.109	.256^*^	.276^**^	.032	-.121	.140	.012	.243^*^
	Sig. (2-tailed)	.678	.	.001	.130	.083	.235	.452	.520	.009	.292	.012	.006	.754	.242	.172	.907	.017
IL-2	Correlation coefficient (ρ)	.141	.343^**^	1.000	.469^**^	.185	.330^**^	-.103	.118	.287^**^	.133	.299^**^	.319^**^	.156	-.002	.071	.248^*^	.090
	Sig. (2-tailed)	.172	.001	.	.000	.071	.001	.319	.252	.005	.196	.003	.002	.130	.984	.491	.015	.384
IL-4	Correlation coefficient (ρ)	.228^*^	.156	.469^**^	1.000	.262^*^	.358^**^	.119	-.015	.176	.280^**^	.190	.109	.320^**^	.279^**^	.240^*^	.377^**^	.156
	Sig. (2-tailed)	.025	.130	.000	.	.010	.000	.248	.885	.087	.006	.064	.289	.001	.006	.019	.000	.130
IL-5	Correlation coefficient (ρ)	.212^*^	.178	.185	.262^*^	1.000	.269^**^	.054	.092	.323^**^	.213^*^	.178	-.015	.248^*^	.432^**^	.211^*^	.129	.377^**^
	Sig. (2-tailed)	.038	.083	.071	.010	.	.008	.600	.373	.001	.038	.082	.886	.015	.000	.039	.210	.000
IL-6	Correlation coefficient	.275^**^	.122	.330^**^	.358^**^	.269^**^	1.000	.125	.334^**^	.301^**^	.118	.230^*^	.058	.241^*^	.274^**^	.366^**^	.223^*^	.244^*^
	Sig. (2-tailed)	.007	.235	.001	.000	.008	.	.225	.001	.003	.253	.024	.572	.018	.007	.000	.029	.017
IL-7	Correlation coefficient (ρ)	.196	-.078	-.103	.119	.054	.125	1.000	-.042	.075	.151	.174	-.118	.293^**^	.222^*^	.247^*^	.169	-.040
	Sig. (2-tailed)	.056	.452	.319	.248	.600	.225	.	.688	.465	.143	.089	.253	.004	.030	.015	.099	.702
IL-8	Correlation coefficient (ρ)	.100	.066	.118	-.015	.092	.334^**^	-.042	1.000	.128	.124	-.042	.047	.167	.088	.277^**^	-.058	.378^**^
	Sig. (2-tailed)	.332	.520	.252	.885	.373	.001	.688	.	.213	.230	.687	.647	.105	.394	.006	.576	.000
IL-10	Correlation coefficient (ρ)	.279^**^	.264^**^	.287^**^	.176	.323^**^	.301^**^	.075	.128	1.000	.392^**^	.367^**^	.382^**^	.043	.102	.355^**^	.093	.141
	Sig. (2-tailed)	.006	.009	.005	.087	.001	.003	.465	.213	.	.000	.000	.000	.679	.324	.000	.366	.172
IL-12	Correlation coefficient (ρ)	.068	.109	.133	.280^**^	.213^*^	.118	.151	.124	.392^**^	1.000	.201^*^	.076	.309^**^	.227^*^	.084	.244^*^	.255^*^
	Sig. (2-tailed)	.507	.292	.196	.006	.038	.253	.143	.230	.000	.	.050	.461	.002	.026	.417	.016	.012
IL-13	Correlation coefficient (ρ)	.136	.256^*^	.299^**^	.190	.178	.230^*^	.174	-.042	.367^**^	.201^*^	1.000	.198	.225^*^	.157	.296^**^	.326^**^	.096
	Sig. (2-tailed)	.186	.012	.003	.064	.082	.024	.089	.687	.000	.050	.	.053	.027	.126	.003	.001	.353
IL-17	Correlation coefficient (ρ)	.061	.276^**^	.319^**^	.109	-.015	.058	-.118	.047	.382^**^	.076	.198	1.000	-.217^*^	-.140	.348^**^	-.037	-.134
	Sig. (2-tailed)	.557	.006	.002	.289	.886	.572	.253	.647	.000	.461	.053	.	.034	.173	.001	.719	.192
G-CSF	Correlation coefficient (ρ)	.030	.032	.156	.320^**^	.248^*^	.241^*^	.293^**^	.167	.043	.309^**^	.225^*^	-.217^*^	1.000	.480^**^	.278^**^	.229^*^	.353^**^
	Sig. (2-tailed)	.775	.754	.130	.001	.015	.018	.004	.105	.679	.002	.027	.034	.	.000	.006	.025	.000
GM-CSF	Correlation coefficient (ρ)	.190	-.121	-.002	.279^**^	.432^**^	.274^**^	.222^*^	.088	.102	.227^*^	.157	-.140	.480^**^	1.000	.290^**^	.205^*^	.327^**^
	Sig. (2-tailed)	.063	.242	.984	.006	.000	.007	.030	.394	.324	.026	.126	.173	.000	.	.004	.045	.001
TNF-α	Correlation coefficient (ρ)	.247^*^	.140	.071	.240^*^	.211^*^	.366^**^	.247^*^	.277^**^	.355^**^	.084	.296^**^	.348^**^	.278^**^	.290^**^	1.000	.039	.285^**^
	Sig. (2-tailed)	.015	.172	.491	.019	.039	.000	.015	.006	.000	.417	.003	.001	.006	.004	.	.705	.005
TNF-β	Correlation coefficient (ρ)	.239^*^	.012	.248^*^	.377^**^	.129	.223^*^	.169	-.058	.093	.244^*^	.326^**^	-.037	.229^*^	.205^*^	.039	1.000	-.149
	Sig. (2-tailed)	.019	.907	.015	.000	.210	.029	.099	.576	.366	.016	.001	.719	.025	.045	.705	.	.147
MCP-1	Correlation coefficient (ρ)	-.004	.243^*^	.090	.156	.377^**^	.244^*^	-.040	.378^**^	.141	.255^*^	.096	-.134	.353^**^	.327^**^	.285^**^	-.149	1.000
	Sig. (2-tailed)	.971	.017	.384	.130	.000	.017	.702	.000	.172	.012	.353	.192	.000	.001	.005	.147	.
